# P-317. Performance of nasal MRSA PCR screening test in pediatric patients admitted to hospital

**DOI:** 10.1093/ofid/ofae631.520

**Published:** 2025-01-29

**Authors:** Rima Shrestha, Amandeep Sehra, Natalia Gawelda, Mustafa Bakir

**Affiliations:** University of Illinois College of Medicine at Peoria, Peoria, Illinois; University of Illinois College of Medicine at Peoria, Peoria, Illinois; University of Illinois College of Medicine at Peoria, Peoria, Illinois; University of Illinois College of Medicine at Peoria, Peoria, Illinois

## Abstract

**Background:**

Few studies reported Methicillin-Resistant Staphylococcus aureus (MRSA) polymerase chain reaction (PCR) nasal screening as a tool for clinical stewardship in children.

This multicenter study evaluated the diagnostic test characteristics of the MRSA-PCR in predicting the presence of invasive MRSA infection in large number of pediatric patients admitted to hospital.

**Figure:**
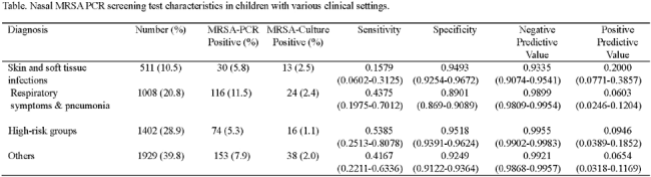

**Methods:**

A retrospective cohort study is performed by chart review of children over 1 month of age admitted to OSF Health Care System, Illinois, between May 2013 and June 2022 and who has at least one MRSA-PCR nasal screening test and any subsequent culture of sterile body site performed. Data were validated, duplicates were removed, and missing or incomplete information related to screening results were removed before predictive values and post-test probabilities were calculated for MRSA infection in all patients as well as subgroup of high-risk, skin-soft tissue infections, and patients who were presented with respiratory symptoms or diagnosed with pneumonia.

**Results:**

Overall, 373 (7.7%) of 4850 patients were tested positive with MRSA-PCR. Among patients screened positive, 91 (1.9%) had invasive MRSA infection. Sensitivity, specificity, positive and negative predictive value of screening test are 32.9%, 92.8%, 98.6%, and 8.0%, respectively. Table shows the screening test characteristics for various clinical diagnoses.

**Conclusion:**

Large-scale nasal PCR screening test for MRSA in hospital-admitted pediatric patients shows very high negative predictive value, particularly in high-risk group. However, low prevalence of MRSA infection may not allow this screening tool to de-escalate antibiotic coverage in children admitted to hospital and suspected of invasive MRSA infection.

**Disclosures:**

**All Authors**: No reported disclosures

